# Hub genes, diagnostic model, and predicted drugs in systemic sclerosis by integrated bioinformatics analysis

**DOI:** 10.3389/fgene.2023.1202561

**Published:** 2023-07-12

**Authors:** Yue-Mei Yan, Meng-Zhu Jin, Sheng-Hua Li, Yun Wu, Qiang Wang, Fei-Fei Hu, Chen Shen, Wen-Hao Yin

**Affiliations:** ^1^ Department of Dermatology, The First Hospital of Jiaxing, The Affiliated Hospital of Jiaxing University, Jiaxing, China; ^2^ Department of Dermatology, Zhongshan Hospital, Fudan University, Shanghai, China; ^3^ Department of Dermatology, Shanghai Skin Disease Hospital, Tongji University, Shanghai, China

**Keywords:** systemic sclerosis, weighted gene co-expression network analysis, connectivity map, molecular docking, phosphodiesterase inhibitors

## Abstract

**Background:** Systemic sclerosis (scleroderma; SSc), a rare and heterogeneous connective tissue disease, remains unclear in terms of its underlying causative genes and effective therapeutic approaches. The purpose of the present study was to identify hub genes, diagnostic markers and explore potential small-molecule drugs of SSc.

**Methods:** The cohorts of data used in this study were downloaded from the Gene Expression Complex (GEO) database. Integrated bioinformatic tools were utilized for exploration, including Weighted Gene Co-Expression Network Analysis (WGCNA), least absolute shrinkage and selection operator (LASSO) regression, gene set enrichment analysis (GSEA), Connectivity Map (CMap) analysis, molecular docking, and pharmacokinetic/toxicity properties exploration.

**Results:** Seven hub genes (THY1, SULF1, PRSS23, COL5A2, NNMT, SLCO2B1, and TIMP1) were obtained in the merged gene expression profiles of GSE45485 and GSE76885. GSEA results have shown that they are associated with autoimmune diseases, microorganism infections, inflammatory related pathways, immune responses, and fibrosis process. Among them, THY1 and SULF1 were identified as diagnostic markers and validated in skin samples from GSE32413, GSE95065, GSE58095 and GSE125362. Finally, ten small-molecule drugs with potential therapeutic effects were identified, mainly including phosphodiesterase (PDE) inhibitors (BRL-50481, dipyridamole), TGF-β receptor inhibitor (SB-525334), and so on.

**Conclusion:** This study provides new sights into a deeper understanding the molecular mechanisms in the pathogenesis of SSc. More importantly, the results may offer promising clues for further experimental studies and novel treatment strategies.

## Introduction

Systemic sclerosis (scleroderma; SSc) is a heterogeneous connective tissue disease characterized by progressive cutaneous and visceral fibrosis (Denton and Khanna, 2017). The overall prevalence and incidence of SSc in global were 17.6 per 100, 000 and 1.4 per 100,000 person-years, respectively (Bairkdar et al., 2021). Though uncommon, SSc has the highest cause-specific mortality among all the rheumatic diseases, and main causes of death are lung and heart involvement (Tyndall et al., 2010; Elhai et al., 2017). Also, SSc imposes high burden in terms of life quality of the patients and social cost.

Fibroproliferative vasculopathy and pronounced immunity abnormalities are involved in the onset and etiopathogenesis of SSc, ultimately leading to the irreversible fibrosis development––the typical hallmark in SSc. In the process of fibroproliferation, transforming growth factor-β (TGF-β) plays a pivotal role while multiple cytokines have been implicated, such as connective tissue growth factor (CTGF), interleukin (IL), chemokines and so on (Clark, 1985; Hu et al., 2018). However, the subtle mechanisms underpinning clinical heterogeneity are, by far, poorly understood. Early differentiation and diagnosis, with commencement of modifying treatment, aids to improve the outcomes in patients with SSc (Jerjen et al., 2022). Therefore, improved understanding of the pathophysiology of SSc is required to provide new strategies for the diagnosis and treatment of SSc.

Public databases combined with bioinformatics tools provide novel insights on elucidating the potential mechanisms and promising biomarkers (The Gene Ontology Consortium, 2019). Weighted gene co-expression network analysis (WGCNA) is an important method to understand gene function and gene association from the genetic level (Langfelder and Horvath, 2008). Least absolute shrinkage and selection operator (LASSO) is a regression-based methodology identifying regression coefficients for genes to shrink a weighted average of mean squared prediction error for cases (Langfelder and Horvath, 2008). Drug database also provide us a good opportunity to discover new therapeutic strategies to reverse disease progression.

In this investigation, we aimed to explore the hub genes and diagnostic markers related to the disease course, and further seek for new drugs for the treatment of SSc. Microarray datasets of SSc retrieved from the NCBI Gene Expression Omnibus public database (GEO) datasets were utilized for discovery and validation. Hub genes were identified, and a diagnostic model was created based on the WGCNA algorithm and machine-learning technique. Moreover, to the best of our knowledge, small-molecule compounds for the treatment of SSc were predicted using the Connectivity Map (CMap) analysis for the first time. Our findings may cast novel sights into the better understanding the pathogenesis of SSc and point to the potential drugs for accurate therapy of SSc.

## Materials and methods

### Data collection and preprocessing

Gene expression profiling datasets were obtained from the NCBI Gene Expression Omnibus public database (GEO) (https://www.ncbi.nlm.nih.gov/geo/). Screening was performed in accordance with the following criteria: 1) Tissues originate from skin biopsy on *Homo sapiens*; 2) At least 10 samples were included; 3) Samples have been treated with no modifying drugs. Finally, the GEO dataset numbered GSE45485, GSE76885, GSE32413, GSE95065, GSE58095, and GSE125362 were selected.

Individual datasets underwent stringent quality control, background correction, log2 transformation, and normalization in the environment of the R software (version 4.2.1). Agilent microarrays (GSE45485, GSE76885, GSE125362, and GSE32413) were normalized using the “limma” package; Illumina microarray (GSE58095) was normalized using the “lumi” package; Affymetrix microarray (GSE95065) was subjected to RMA normalization using the “affy” package, respectively. GSE45485 and GSE76885 were merged, and the batch effects were corrected with the ComBat function of the “sva” package in R. A total of 135 samples (38 HCs and 97 SSc patients) of the merged GSE45485 and GSE76885 were utilized to conduct the WGCNA analysis. And GSE32413, GSE95065, GSE58095, and GSE125362 were utilized for the validation, respectively.

### Differential expression analysis

Differential expression analysis of HC and SSc samples was performed using the “limma” package. With |log2 fold change (FC)| > 0.585 and adjusted *p* < 0.05 as the cutoff threshold, differentially expression genes (DEGs) were detected. To better visualize the results, heatmap and volcano plot of DEGs were generated using the “pheatmap” and “ggplot2” packages.

### Construction of a weighted gene co-expression network

To investigate the co-expression relationships among the genes and the relationship between the genes and the phenotypes, weighted correlation network analysis (WGCNA) method was applied using the “WGCNA” package in R. After filtering the outlier samples, with an optimum soft threshold was set, the weighted adjacency matrix was transformed into a topological overlap matrix (TOM) to estimate the network connectivity. Then, the co-expression modules were clustered by a dynamic tree-cut approach on TOM-based dissimilarity. Genes with similar patterns were grouped into a module. At last, correlation coefficient analysis of module membership (MM) with gene significance (GS) was implemented.

### Identification of hub genes

After identifying the key module that most representing the SSc disease trait, the intra-module connectivity (IMConn) was then calculated to determine the top 30 genes with the highest connectivity within the key module. Besides, the criteria (absolute values of GS > 0.45 and MM > 0.80) was used to screen the genes with biological importance in the key module. The intersection of DEGs, top 30 genes with the highest IMConn, and genes with biological importance in the key module, was taken using the tool on Evenn website (http://www.ehbio.com/test/venn/). The common genes were defined as the final hub genes of SSc.

### Functional enrichment analysis

The functional annotation of DEGs analyzed by Gene Ontology (GO) was reflected in biological processes (BP), cell components (CC), molecular function (MF). Kyoto Encyclopedia of Genes and Genomes (KEGG) analysis was performed to analyze related significant pathways. The GO and KEGG pathways were retrieved with a cut-off criterion of *p* < 0.05 and visualized by the “enrichplot” and “GOplot” packages in R.

The respective functions of each hub gene were revealed by Gene Set Enrichment Analysis (GSEA). After removing HC samples, the left SSc samples were distinguished into two groups, the low expression group and the high expression group, based on median values of hub gene expression levels. Differential expression analysis between the two groups was performed and genes were sorted by logFC from the highest to the lowest. The ridge plots were presented using the “clusterprofiler” and “enrichplot” package in R.

### Screening and validation of diagnostic model

To identify the diagnostic markers among hub genes, LASSO regression was conducted using the “lars” package in R. Next, the sensitivity and specificity of the diagnostic model were evaluated using receiver operating characteristic curves (ROCs) using the “pROC” package in R. The diagnostic performance of the model was assessed by the area under the curve (AUC), and AUC > 0.75 was set as the cut-off value. In general, the gene expression profile of the merged dataset (GSE45485 and GSE76885, n = 135) was used as discovery cohort, and the gene expression profiles of GSE32413 (n = 35), GSE95065 (n = 33), GSE58095 (n = 102), and GSE125362 (n = 12) were used as validation cohorts to verify the ability of diagnostic model. The correlations between diagnostic markers and well-known causative genes (TGFB1, CTGF, COL1A1, COL1A2, IL6, CCL2, VCAM1, and THBS1) in the merged dataset were assessed by Pearson’s correlation test using the “ggplot2” package in R.

### Drug prediction

Connectivity Map (CMap) is a collection of databases that stores a pool of gene transcription-expression profiles from cultured mammalian cells exposed to active small molecule drugs. Top 30 genes with the highest IMConn were uploaded to the L1000 platform (https://clue.io/) for prediction of potential drugs towards SSc for pharmaceutical development. Compounds with the CMap negative connectivity score of −90 or lower, indicating higher potential anti-SSc effect, were considered to be potential effective drugs. Meanwhile, SwissTargetPrediction online database (http://www.swisstargetprediction.ch/) was utilized to predict the targets of the predicted drugs.

The molecular structures of predicted compounds were obtained from PubChem Compound (https://pubchem.ncbi.nlm.nih.gov/). The 3D coordinates of predicted targets were downloaded from the PDB (http://www.rcsb.org/pdb/home/home.do). AutoDockTools (version 1.5.7), AutoDock Vina (version 1.1.2), PyMOL (version 2.5) and ChemDraw (version 19.0) softwares were utilized for molecular docking studies and model visualization, respectively. The pkCSM (http://structure.bioc.cam.ac.uk/pkcsm) server was utilized to measure ADMET (absorption, distribution, metabolism, excretion, and toxicity) properties of predicted compounds (Pires et al., 2015). Using the canonical smiles strings retrieved from the PubChem database, pharmacokinetic and toxicity properties were calculated.

### Statistical analysis

All statistical analyses were performed using R software. *p* < 0.05 was considered statistically significant.

## Results

### Identification and functional enrichment analysis of DEGs

**FIGURE 1:**
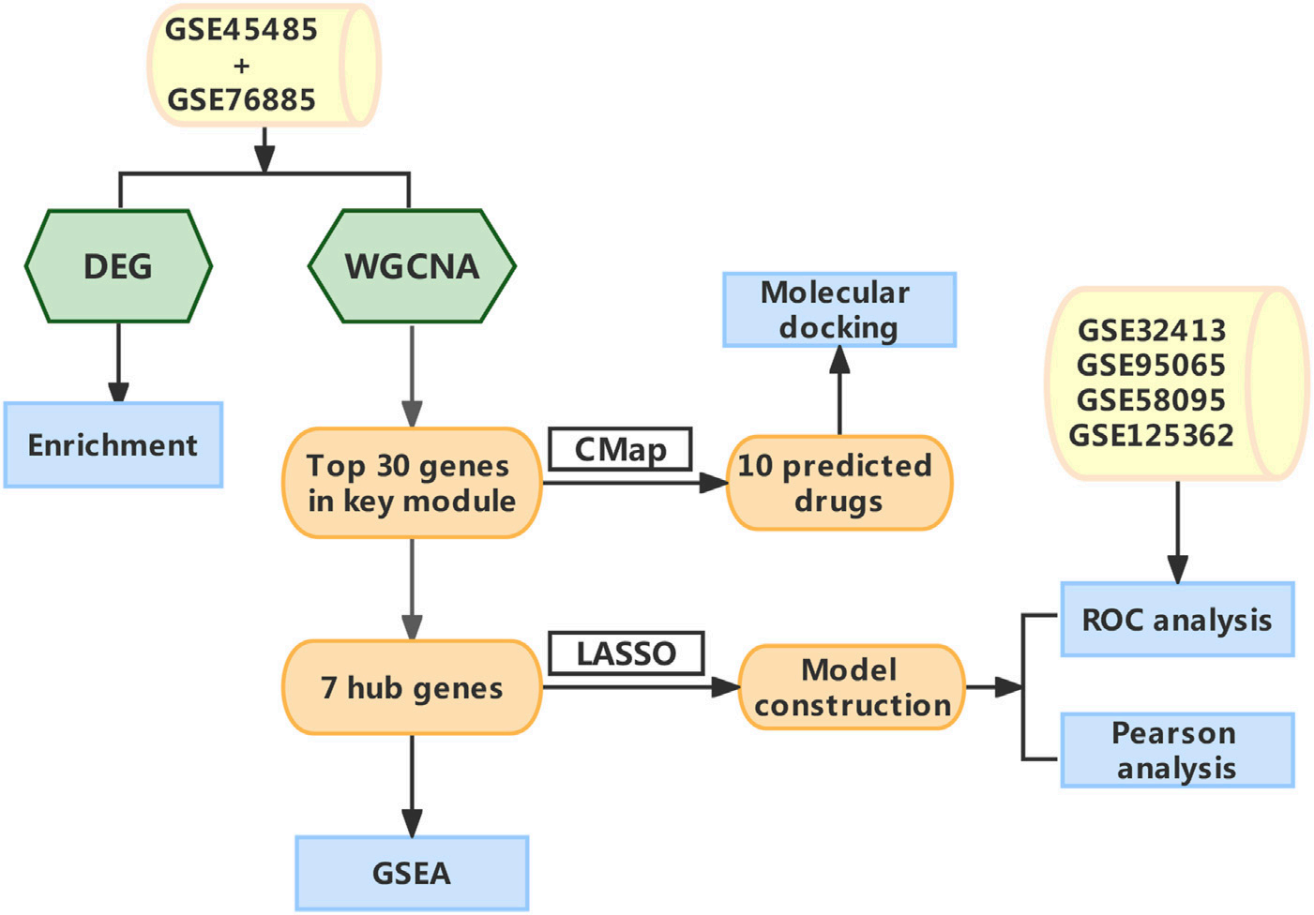
The schematic diagram of the data Analysis. Abbreviations: DEG, differentially expressed genes; WGCNA, Weighted gene co-expression network analysis; CMap, Connectivity map; LASSO, least absolute shrinkage and selection operator; GSEA, gene set enrichment analysis; ROC, receiver operating characteristic.

**FIGURE 2:**
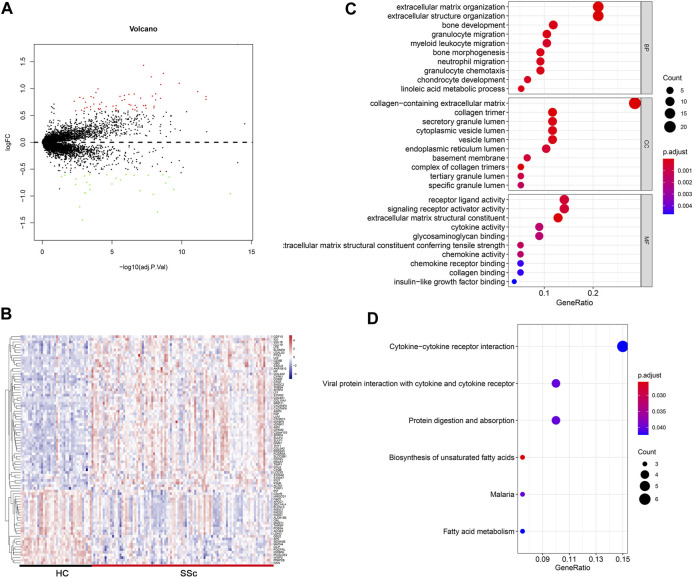
Differentially expressed gene profiles analysis. **(A,B)** Volcano plot and heatmap of DEGs between HC and SSc skin samples in the merged gene expression profile of GSE845485 and GSE76885. **(C,D)** Top 10 biological process (BP), cellular component (CC), molecular functions (MF) terms and top 6 KEGG enrichment pathways of DEGs in the merged gene expression profile of GSE845485 and GSE76885. The size of each circle means the amounts of genes. The different color of each circle means p-adjust-value. GeneRatio means the ratio of genes that belong to this pathway divided by the number of genes in the background gene cluster that belong to this pathway. Abbreviations: DEG: differentially expressed genes; HC, healthy control; KEGG: Kyoto Encyclopedia of Genes and Genomes.

The flow chart of the study was summarized in Figure 1. Details of the collected datasets are presented in Table 1. The DEGs were investigated in HC and SSc in the merged microarray dataset (GSE45485 and GSE76885). A total of 86 genes were identified to be differentially expressed between HC and SSc samples, of which 58 genes were upregulated and 28 genes were downregulated. The volcano plot and heatmap of DEGs in each group were presented in Figures 2A, B.

**TABLE 1:** Detailed information of GEO datasets.

GSE number	Platform		Samples (HC vs. SSc)	Tissue
GSE45485	GPL6480	Agilent-014850	20 vs. 33	Skin
GSE76885	GPL6480	Agilent-014850	18 vs. 64	Skin
GSE125362	GPL6480	Agilent-014850	4 vs. 8	Skin
GSE95065	GPL23080	Affymetrix [HG-U133A_2]	15 vs. 18	Skin
GSE58095	GPL10558	Illumina HumanHT-12 V4.0	43 vs. 59	Skin
GSE32413	GPL4133	Agilent-014850	8 vs. 27	Skin

HC: healthy control; SSc: systemic sclerosis.

To gain insights into the biological roles of the DEGs, we performed GO categories enrichment analysis. With the criterion of *p* < 0.05, “extracellular matrix organization”, “extracellular structure organization”, “bone development”, “granulocyte migration”, and “myeloid leukocyte migration” exhibited highly significant enrichment within the BP category. For the CC category, DEGs were significantly enriched in “collagen-containing extracellular matrix”, “collagen timer”, “secretory granule lumen”, “cytoplasmic vesicle lumen”, and “vesicle lumen”. In addition, the MF category contained DEGs significantly enriched in “receptor ligand activity”, “signaling receptor activator activity”, “extracellular matrix structural constituent”, “cytokine activity” and “glycosaminoglycan binding” (Figure 2C). The top enriched KEGG pathways included “cytokine–cytokine receptor interaction”, “viral protein interaction with cytokine and cytokine receptor”, “protein digestion and absorption”, and “biosynthesis of unsaturated fatty acids” (Figure 2D).

### Construction of co-expression modules

**FIGURE 3:**
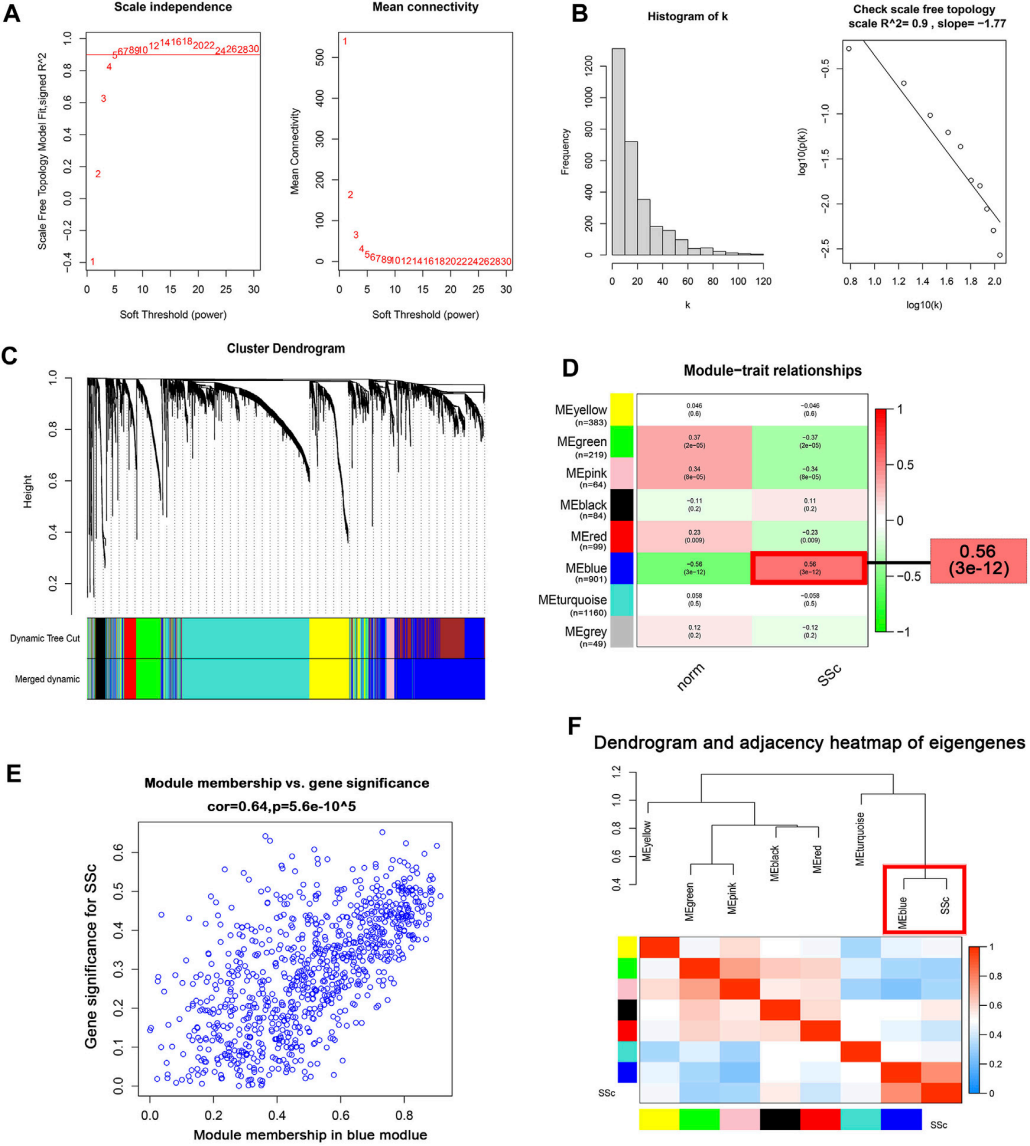
Construction of the co-expression network. **(A)** Analysis of the scale-free topology model fit index for soft threshold powers (*β*) and the mean connectivity for soft threshold powers. **(B )** Histogram of the connectivity distribution and check scale-free topology scale when the soft threshold power (*β*) was 5. **(C )** Cluster dendrogram of the co-expression network modules was produced based on topological overlap in the merged gene expression profiles of GSE845485 and GSE76885. Each branch means one gene; Each color means one co-expressed module. **(D)** Heatmap of the correlation between module eigengenes and clinical traits. Red represents a positive correlation, and blue represents a negative correlation. **(E)** Correlation of module membership (MM) and gene significance (GS) in the MEblue module. **(F)** Dendrogram and unsupervised hierarchical clustering heatmap of module eigengenes and SSc. Abbreviations: SSc: systemic sclerosis; MM, module membership; GS, gene significance.

WGCNA algorithm was used to identify the co-expressed genes and modules in the merged gene expression datasets of GSE45485 and GSE76885. To construct the scale-free clustering dendrograms, soft threshold power was picked as 5 (Figure 3A). The signed R2 was shown in a log-log linear model for module connectivity analysis is R2 = 0.90, suggesting the successful construction of scale-free correlation (Figure 3B). After merging similar modules, eight modules with high adjacency from the co-expression network were visualized in the dendrograms (Figure 3C). According to the module-trait relationships, the MEblue module was identified as the key module that most associated with SSc disease trait (Cor = 0.64, *p* < 0.01) (Figures 3D, E). The dendrogram and adjacency heatmap of eigengenes further indicated that the MEblue module was the closest one to reflect the pathogenesis of SSc (Figure 3F). Thus, MEblue module was selected for downstream analysis.

### Identification and GSEA of hub genes

**FIGURE 4:**
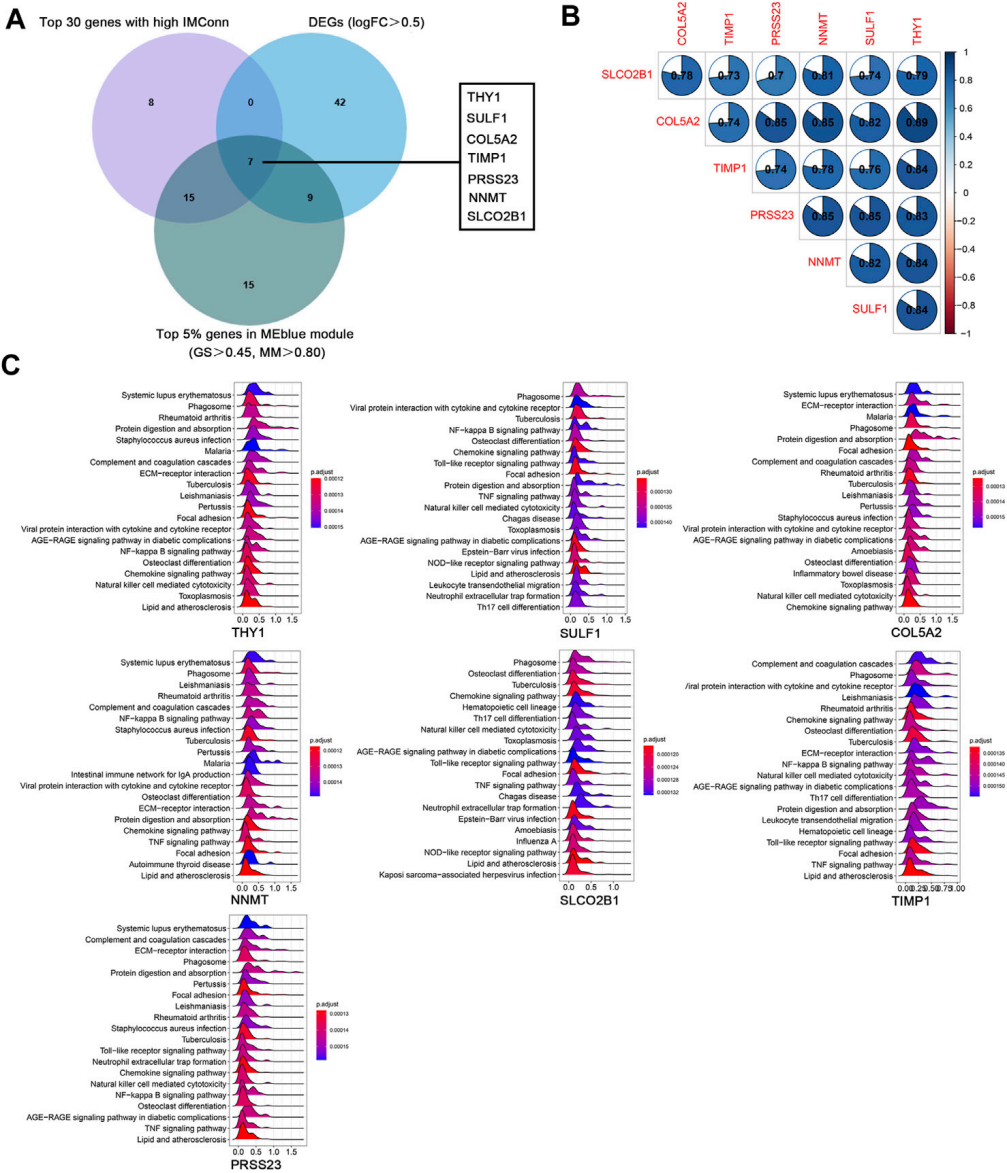
Hub genes and GSEA. **(A)** seven hub genes were obtained by taking the intersections of 58 upregulated genes, top 5% genes (GS > 0.45, MM > 0.80) in the MEblue module, and top 30 genes with the highest IMConn in the MEblue module. **(B )** Correlation analysis among seven hub genes revealed that they were highly connected in the expression levels with each other. **(C)** GSEA revealed the enriched pathways of THY1, SULF1, COL5A2, NNMT, SLCO2B1, TIMP1, and PRSS23. Abbreviations: GS, gene significance; MM, module membership; IMConn, intra-module connectivity; GSEA, gene set enrichment analysis.

A total of 901 genes were included in the MEblue model. On one hand, with GS > 0.45 and MM > 0.80, 45 genes (top 5% of all genes in the key module) with biological importance in the MEblue module were filtered out. On the other hand, on the basis of the expression values of IMConn, the top 30 highly connected genes in the key module to mine potential key molecules were selected. Venn plot showed the intersection of DEGs, top 30 genes with the highest IMConn, and 45 genes with biological importance (Figure 4A). Thus, 7 overlapping genes (THY1, SULF1, PRSS23, COL5A2, NNMT, SLCO2B1, and TIMP1) were ultimately selected and defined as the final hub genes that might be involved in the pathogenesis of SSc. Detailed information of hub genes was presented in Table 2. Correlation analysis among 7 hub genes revealed that they were highly connected in the expression levels with each other (Figure 4B).

**TABLE 2:** Hub genes.

	Gene name	IMConn	GS	MM	LogFC
1	COL5A2	82.43	0.54	0.88	0.64
2	NNMT	80.55	0.47	0.88	0.87
3	SLCO2B1	87.98	0.49	0.91	0.65
4	PRSS23	83.97	0.53	0.88	0.86
5	THY1	86.10	0.62	0.90	0.94
6	SULF1	69.90	0.56	0.84	1.28
7	TIMP1	70.44	0.50	0.85	0.69

IMConn: Intra-module connectivity; GS: gene significance; MM: module membership; LogFC: log fold change. The *p* values of GS, MM, LogFC are all <0.01.

GSEA results revealed the potential biological roles of hub genes (Figure 4C). The ridge plots have shown that they are associated with autoimmune diseases (systemic lupus erythematosus, rheumatoid arthritis, and lipid and atherosclerosis), microorganism infections (tuberculosis, *staphylococcus aureus* infection, pertussis, and leishmaniasis), inflammatory related pathways (NF-kappa B signaling pathway, chemokine signaling pathway, TNF signaling pathway, AGE-RAGE signaling pathway in diabetic complications), immune responses (phagosome, complement and coagulation cascades, natural killer cell mediated cytotoxicity), and fibrosis process (protein digestion and absorption, ECM-receptor interaction, focal adhesion, osteoclast differentiation).

### Construction and validation of a diagnostic model

**FIGURE 5:**
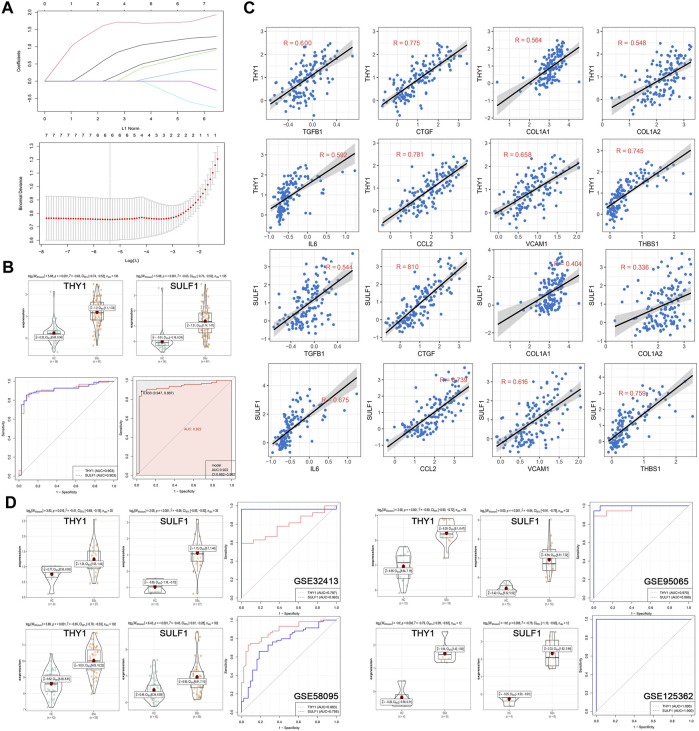
Establishment and validation of diagnostic biomarkers. **(A)** LASSO coefficient profiles of the seven genes in SSc. The log(lambda) sequence was used to construct a coefficient profile diagram. The LASSO model’s optimal parameter (lambda) was chosen. **(B)** Expression of THY1 and SULF1 in the HC and SSc skin samples in the merged gene expression profiles of GSE45485 and GSE76885. ROC curves and corresponding AUC values of THY1, SULF1, and combination of them in the merged gene expression profiles of GSE45485 and GSE76885. **(C)** The relationships between the diagnostic markers and causative genes (TGFB1, CTGF, COL1A1, COL1A2, IL6, CCL2, VCAM1, and THBS1) was verified by Pearson correlation analysis in the merged gene expression profiles of GSE45485 and GSE76885. **(D)** Expression of THY1 and SULF1 in the HC and SSc skin samples in validation cohorts (GSE32413, GSE95065, GSE58095 and GSE125362, respectively). ROC curves and corresponding AUC values for the four expression cohorts. Abbreviations: HC, healthy control; SSc, systemic sclerosis; LASSO, least absolute shrinkage and selection operator; ROC, receiver operating characteristic; AUC, area under the curve.

In the discovery cohort of GSE45485 and GSE76885, LASSO regression algorithm was employed to further screen prognostic SSc-related signature genes. According to the minimum partial likelihood deviance and optimum *λ* value, THY1 and SULF1 were identified as prognostic markers (Figure 5A). The violin plots revealed that THY1 and SULF1 exhibited higher expression levels in SSc patients than HCs (*p* < 0.05) (Figure 5B). The AUC values of THY1 and SULF1 in the merged dataset were 0.903, 0.903, respectively, suggesting high diagnostic efficacy, and the combined diagnostic value of THY1 and SULF1was 0.922 (Figure 5B). Then, the relationships between the diagnostic markers and causative genes (TGFB1, CTGF, COL1A1, COL1A2, IL6, CCL2, VCAM1, and THBS1) was verified by Pearson correlation analysis. The expression levels of THY1 and SULF1 were positively associated with these causative genes levels with high relevance (*p* < 0.05) (Figure 5C). To further verify the diagnostic markers, expression level detection and ROC analysis were conducted in the validation datasets. The expression levels of THY1 and SULF1 were higher in SSc patients than HCs, based on the violin plots of GSE32413, GSE95065, GSE58095 and GSE125362 (*p* < 0.05) (Figure 5D). Similarly, the AUC values of THY1 and SULF1 were 0.787, 0.963 in GSE32413, respectively; 0.970, 0.996 in GSE95065, respectively; 0.883, 0756 in GSE58095, respectively; and 1.000, 1.000 in GSE125362, respectively (Figure 5D).

### Drug prediction for SSc

**FIGURE 6:**
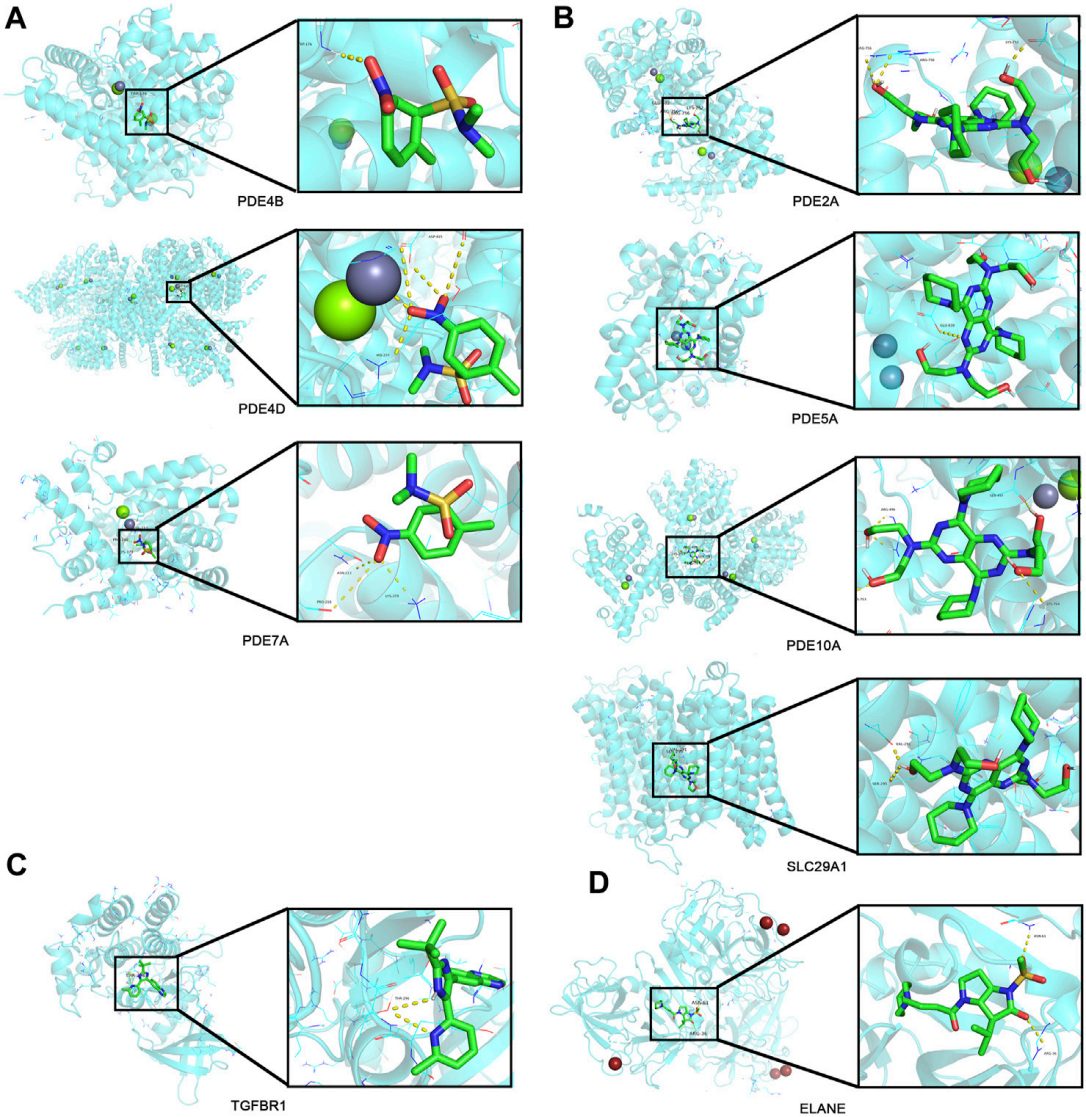
Drug prediction and molecular docking. **(A)** Molecular docking analyses for BRL-50481 with target proteins PDE4B, PDE4D, and PDE7A, respectively. **(B)** Molecular docking analyses for dipyridamole with target proteins PDE2A, PDE5A, PDE10A, and SLC29A1, respectively. **(C)** Molecular docking analyses for SB-525334 with target protein TGFBR1. **(D)** Molecular docking analyses for GW-311616 with target protein ELANE.

Top 30 genes with the highest IMConn were then incorporated into the CMap database for further analysis to screen target drugs related to SSc. According to CMap score, top 10 small-molecule drugs with potential therapeutic effects were ranked as follows: desoxypeganine, clofazimine, BRL-50481, GW-311616, methyllycaconitine, acetyl-geranyl-cysteine, SB-525334, dipyridamole, tomelukast, and warfarin (Table 3). The 3D structure diagrams of these candidate molecule drugs are shown in Supplementary Figure S1. The targets of the predicted drugs analyzed by SwissTargetPrediction online database were also summarized in Table 3. Molecular docking analysis was based on the structure of proteins and structure of drugs. It is believed that the molecular docking binding energy is less than 0, indicating that the ligand and the receptor can spontaneously bind. The results showed that BRL-50481 bounds to PDE4B, PDE4D, and PDE7A with the low binding energy of −4.5, −5.8, and −4.8 kcal/mol, respectively (Figure 6A). Similarly, dipyridamole bounds to PDE2A, PDE5A, PDE10A, SLC29A1 with the low binding energy of −5.5, −4.5, −5.4 and −5.2 kcal/mol, respectively (Figure 6B). SB-525334 bonds to TGFBR1 with the low binding energy of −6.4 kcal/mol (Figure 6C). And, GW-311616 bonds to ELANE with the low binding energy of −5.7 kcal/mol (Figure 6D). These data all indicated the highly stable binding between drugs and proteins.

**TABLE 3:** Top 10 chemical compounds identified by L1000 platform.

Rank	Compound name	PubChem CID	CMap score	Description	Targets (SwissTargetPredic-tion Probability>0.95)
1	desoxypeganine	442,894	−99.93	Acetylcholinesterase inhibitor	/
2	clofazimine	2794	−99.93	GK0582 inhibitor	/
3	BRL-50481	2921148	−99.89	Phosphodiesterase inhibitor	PDE4B, PDE4D, PDE7A
4	GW-311616	9800961	−99.89	Leukocyte elastase inhibitor	ELANE
5	methyllycaconitine	5288811	−99.89	Acetylcholine receptor antagonist	/
6	acetyl-geranyl-cysteine	87288217	−99.89	Isoprenylated protein methylation inhibitor	/
7	SB-525334	9967941	−99.89	TGF beta receptor inhibitor	TGFBR1
8	dipyridamole	3108	−99.86	Phosphodiesterase inhibitor	PDE5A, SLC29A1, PRUNE1, PDE2A, PDE11A, PDE10A
9	tomelukast	3969	−99.83	Leukotriene receptor antagonist	/
10	warfarin	54678486	−99.82	Vitamin K antagonist	/

On the other hand, ADMET properties were predicted to further understand how these compounds behave in a biological system, as shown in Table 4. According to the theory provided by the pkCSM server, the compounds are all well absorbed (Intestinal absorption >30%). Dipyridamole is poorly distributed to the brain (log BB < −1) and unable to penetrate central nervous system (log PS < −3), while SB-525334 can readily cross the blood–brain barrier (log BB > 0.3) and penetrate central nervous system (log PS > −2). Mostly, dipyridamole, GW-311616, and BRL-50481 are not inhibitors of cytochrome P, except that BRL-50481 is an inhibitor of CYP1A2. In terms of toxicity, all compounds are predicted to have no skin sensitization, and no obvious cardiotoxicity. However, most of them may have potential AMES toxicity or hepatotoxicity.

**TABLE 4:** Predicted values of ADMET (absorption, distribution, metabolism, excretion, and toxicity) properties of predicted compounds.

Property	Model name	dipyridamole	SB-525334	GW-311616	BRL-50481
Absorption	Water solubility (log mol/L)	−2.927	−2.899	−3.151	−2.403
	Intestinal absorption (human) (%)	66.311	93.366	86.8	84.762
	Skin Permeability (log Kp)	−2.735	−2.735	−3.2	−2.46
Distribution	BBB permeability (log BB)	−1.412	0.374	−0.703	−0.636
	CNS permeability (log PS)	−3.511	−1.783	−3.082	−2.546
Metabolism	CYP1A2 inhibitor	No	Yes	No	Yes
	CYP2C19 inhibitor	No	Yes	No	No
	CYP2C9 inhibitior	No	Yes	No	No
	CYP2D6 inhibitior	No	No	No	No
	CYP3A4 inhibitior	No	Yes	No	No
Excretion	Total Clearance (log mL/min/kg)	0.295	0.798	0.187	1.071
Toxicity	AMES toxicity	No	Yes	Yes	Yes
	Max. tolerated dose (human) (log mg/kg/day)	0.423	0.382	−0.418	0.606
	hERG I inhibitor	No	No	No	No
	hERG II inhibitor	No	Yes	No	No
	Oral Rat Acute Toxicity (LD50) (mol/kg)	1.976	2.476	2.809	2.431
	Hepatotoxicity	Yes	Yes	Yes	No
	Skin Sensitisation	No	No	No	No

## Discussion

Through the combination of multiple microarray data and integrated bioinformatics analysis, seven genes (THY1, SULF1, COL5A2, TIMP1, NNMT, SLCO2B1, and PRSS23) were identified as hub genes that may play vital roles in the pathogenesis of SSc. THY1 and SULF1 were screened as diagnostic markers for the diagnosis of SSc. Furthermore, ten potential small-molecule drugs were predicted by CMap analysis, developing novel strategies for the therapy of SSc.

GSEA analysis of seven hub genes indicated that they were mainly involved in infections, inflammation, autoimmunity, and fibrogenesis, which are roughly in line with previous findings and cognitions of SSc. The etiology or the initial trigger(s) in SSc remains elusive (Fett, 2013). The GSEA results stressed that microbial infections may be considered in the etiology of SSc, or, SSc may initial similar inflammatory and immune responses to microbial infection, including phagosome, complement and coagulation cascades, natural killer cell mediated cytotoxicity and so on. Besides, the results suggested that SSc may share molecular disease pathways, such as the interferon (IFN) type I pathways, with other autoimmune diseases (systemic lupus erythematosus, rheumatoid arthritis) (Ortíz-Fernández et al., 2022). In addition, as an inflammatory fibrotic disease, SSc is related with inflammatory genes including cytokines, chemokines, adhesion molecules and so on. A study found that serum and tissue levels of C-C motif chemokine 2 (CCL2; also known as MCP1), CCL3 (also known as MIP1α), IL-8 and CCL18 are increased in SSc patients and correlate with disease severity and progression (Hasegawa et al., 2013). Inflammatory pathways are also involved in the pathogenesis of SSc. For example, the nuclear factor-κB (NF-κB) pathway regulates the profibrogenic transcriptional programme in fibroblasts and promotes the bleomycin-induced skin fibrosis in mice (Fullard et al., 2013; Worrell et al., 2020).

Two of the seven hub genes, including THY1 and SULF1 were screened to construct a diagnostic model, which may be useful to guide the diagnosis of SSc in clinical applications. THY1 (Thy-1 cell surface antigen; also known as CD90), a 25–37 kDa glycosylphosphatidylinositol (GPI) - anchored glycoprotein, contains an integrin-binding RGD-like motif (RLD). It is implicated in organ fibrosis by regulating the phenotype of fibroblasts and cell-matrix interactions (Rege and Hagood, 2006; Bradley et al., 2009). THY1 was recently found to interact with TGFβRI, indicating a novel mechanism whereby THY1 affects TGF-β1 signalling and myofibroblast differentiation in the contest of liver fibrosis (Koyama et al., 2017). And, by conformational coupling with integrin, THY1 regulates cell adhesion, cytoskeletal organization, and myo-fibroblastic differentiation (Fiore et al., 2015). THY1 expression was markedly elevated in skin and serum in patients with SSc, and co-localized with fibroblast activator protein (FAP) in the deep dermis (Kollert et al., 2013; Marangoni et al., 2022). Thereby, THY1 was identified as a potential biomarker for SSc fibrosis. SULF1 (human sulfatase 1) is a member of sulfatases that hydrolyze sulfate ester bonds of a wide range of substrates while the roles of SULF1 in SSc has been little discussed. A research found that SULF1 over-expression enhances TGF-β and VEGF cell signalling by simultaneously upregulating HS 6-O transferase (HS6ST) activity in dermal endothelial cell (Justo et al., 2022). And, SULF1 may act as an autocrine regulator of fibroblast expansion in the course of an inflammatory response in response to TNF-α stimulation (Sikora et al., 2016). These evidences indicated that SULF1 may be implicated in the pathogenesis of SSc by modulating the activities of growth factors and morphogens. As for others, COL5A2 (collagen type V alpha 2 chain) and TIMP1 (Tissue inhibitor of metalloproteinase 1) regulates ECM deposition and inhibits the ECM degradation (Susol et al., 2000). NNMT (nicotinamide N-methyltransferase) plays a vital role in cancer-associated fibroblasts, involving depletion of S-adenosyl methionine and reduction in histone methylation (Eckert et al., 2019). PRSS23 (serine protease 23), a novel vascular protease, may inhibit the Snail-dependent endothelial-to-mesenchymal transition (EndoMT) to prevent fibrosis (Chen et al., 2013). However, studies on the fibrotic roles of SLCO2B1 (solute carrier organic anion transporter family member 2B1) are rare.

In CMap analysis, it is worth noting that two out of ten predicted drugs are phosphodiesterase (PDE) inhibitors, namely, dipyridamole (targeting PDE5A, SLC29A1, PRUNE1, PDE2A, PDE11A and PDE10A) and BRL-50481 (targeting PDE4B, PDE4D and PDE7A). As vasodilators, PDE inhibitors help to alleviate vasculopathy, the initial event in the pathogenesis of SSc, thereby solving critical ischemia and prevent digital ulcerations (Barsotti et al., 2019). With vasodilator activity, dipyridamole has potent modifying effects in the treatment of progressive SSc patients with thallium-201 myocardial perfusion abnormalities (Kahan et al., 1986). Moreover, dipyridamole may alleviate the pathogenesis of peritoneal fibrosis, involving inhibiting PDGF-stimulated HPMC cell line proliferation and TGF-β-induced collagen gene expression in HPMC, possibly through modulation of the ERK pathway (Hung et al., 2001a; Hung et al., 2001b; Hung et al., 2001c). Recently, more published studies have demonstrated that PDE inhibitors showed good antifibrotic efficacy in various organ fibrosis, especially in lung (Zisman et al., 2010; Brusilovskaya et al., 2020; Richeldi et al., 2022). Evidence showed that PDE inhibitors could reduce skin fibrosis as well. Mirodenafil, a potent PDE5 inhibitor, ameliorated dermal fibrosis in the BLM-induced mice and downregulated the expression of profibrotic genes and collagen in fibroblasts, possibly by suppressing TGF-β/Smad signalling pathway (Roh et al., 2021). Sildenafil, a well-known PDE5 inhibitor, prevents ROS-induced instability in human dermal fibroblasts isolated by SSc patients (Di Luigi et al., 2020). Interestingly, as a PDE4 inhibitor, small-molecule drug apremilast was applied in the treatment of atopic dermatitis, which, like scleroderma, belongs to type 2 inflammatory diseases (Abrouk et al., 2017). Specific inhibition of PDE4 by rolipram and apremilast reduces dermal fibrosis through inhibiting profibrotic cytokines release from M2 macrophages, while fibroblasts are not the direct targets of PDE4 blockade (Maier et al., 2017). Therefore, PDE inhibitors may have therapeutic effects on SSc by alleviating both vasculopathy and fibrosis in skin and lung. As for SB-525334, it is a TGF-β receptor inhibitor as well as an activin receptor-like kinase (ALK5) inhibitor. SB-525334 blocked the expression of fibrotic genes *in vivo*, such as PM2.5-treated hepatocytes, TGF-β1-induced A498 renal epithelial carcinoma cells, and so on (Grygielko et al., 2005; Leilei et al., 2021). SB-525334 treatment significantly attenuated collagen deposition in the bleomycin-induced pulmonary fibrosis and reversed pulmonary arterial pressure by modifying abnormal proliferation of vascular smooth muscle cells (Higashiyama et al., 2007; Thomas et al., 2009). Studies on other drugs are rare. Collectively, dipyridamole, BRL-50481, SB525334 and other predicted drugs are new and promising targets for SSc therapy. However, data mining has its limitations. Even for the same data, using different methods will give different results. Our previous study conducted WGCNA analysis to detect hub genes in GSE58095 and found that serum insulin-like growth factor binding protein 7 (IGFBP7) may be a candidate biomarker for SSc (Yan et al., 2021). Therefore, the effects and molecular mechanisms of the predicted drugs in this study await further experimental validation.

In summary, we identified seven hub genes that may play a pathogenic role through different biological pathways in SSc development. In particular, the diagnostic model of THY1 and SULF1 was created and validated. Moreover, to the best of knowledge, this is the first demonstration that drugs with therapeutic promise for SSc were predicted using CMap analysis. However, our predictions of hub genes, diagnostic model and drugs await further experimental validation in the following studies. Anyway, these findings shed new lights into the development of SSc and may provide therapeutic basis for clinical applications in the prevention of SSc.

## Data Availability

Publicly available datasets were analyzed in this study. This data can be found here: http://www.ncbi.nlm.nih.gov/geo/.
