# Supplementary material for: Hub genes, diagnostic model, and predicted drugs in systemic sclerosis by integrated bioinformatics analysis
Source: Front Genet. 2023 Jul 12;14:1202561. doi: 10.3389/fgene.2023.1202561 (PMC10369177; doi:10.3389/fgene.2023.1202561)
Supplement: Supplementary file 1 [file DataSheet1.PDF]

Supplementary Material

Hub genes, diagnostic model, and predicted drugs in systemic sclerosis by integrated bioinformatics analysis

Yue-Mei Yan[1,2], Meng-Zhu Jin[1], Sheng-Hua Li[1], Yun Wu[1], Qiang Wang[2], Fei-Fei Hu[2], Chen Shen[3], Wen-Hao Yin[1*]

[1] Department of Dermatology, The First Hospital of Jiaxing & The Affiliated Hospital of Jiaxing University, No. 1882 South Zhonghuan Road, Jiaxing City, 314000, Zhejiang, P.R. China.

[2] Department of Dermatology, Zhongshan Hospital, Fudan University, 180 Fenglin Road, Xuhui District, Shanghai 200032, P.R. China

[3] Department of Dermatology, Shanghai Skin Disease Hospital, Tongji University, 1278 Baode Road, Shanghai, 200443, P.R. China.

*** Correspondence:**
Wen-Hao Yin
E-mail: whyin69@sina.com; Tel: +86(573)82519542, Ext.82083006

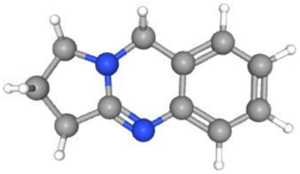

desoxypeganine

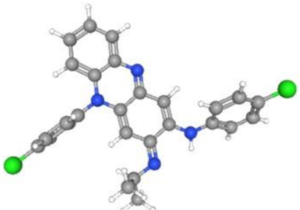

clofazimine

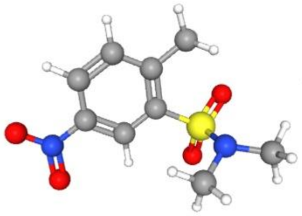

BRL-50481

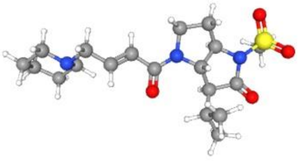

GW-311616

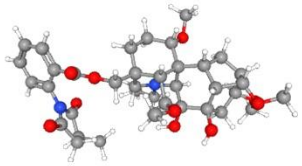

methyllycaconitine

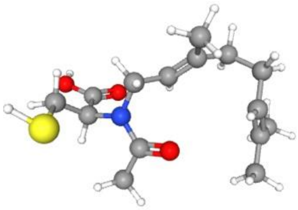

acetyl-geranyl-cysteine

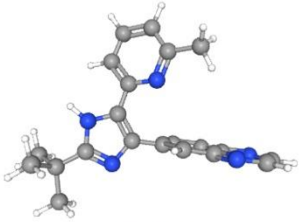

SB-525334

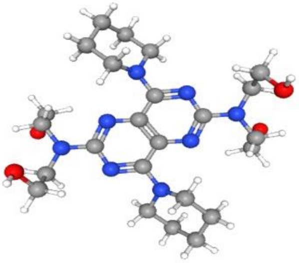

dipyridamole

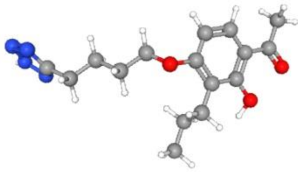

tomelukast

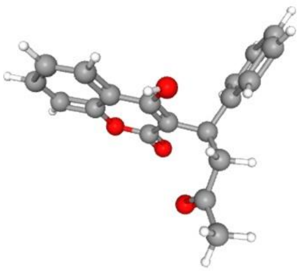

warfarin

Supplementary Figure 1. The structure of predicted drugs.
